# Detection of setup errors with a body‐surface laser‐scanning system for whole‐breast irradiation after breast‐conserving surgery

**DOI:** 10.1002/acm2.13578

**Published:** 2022-03-16

**Authors:** Ping Jiang, Ziyi Liu, Weijuan Jiang, Ang Qu, Haitao Sun, Junjie Wang

**Affiliations:** ^1^ Department of Radiation Oncology Peking University Third Hospital Beijing China

**Keywords:** image‐guided radiotherapy, setup errors, whole‐breast radiotherapy

## Abstract

**Purpose:**

We compared the setup errors determined by an optical imaging system (OSIS) in women who received breast‐conserving surgery (BCS) followed by whole‐breast radiotherapy (WBRT) with those from cone‐beam computed tomography (CBCT) carried out routinely.

**Methods:**

We compared 130 setup errors in 10 patients undergoing WBRT following BCS by analyzing the translational and rotational couch shifts via CBCT and OSIS. Patients were treated with intensity‐modulated radiotherapy (IMRT). The patient outline extracted from the planning reference Computed tomography (CT) was used as the reference for OSIS and CBCT alignment during treatment. We detected the setup uncertainty using CBCT and OSIS at the first five fractionations of RT and then twice a week.

**Results:**

The absolute translational setup error (mean ± Standard deviation (SD)) in x (lateral), y (longitudinal), and z (vertical) axes detected by the OSIS was 0.14 ± 0.18, 0.15 ± 0.14, and 0.13 ± 0.13 cm, respectively. The rotational setup error (mean ± SD) in Rx (pitch), Ry (roll), and Rz (yaw) axes was 0.77 ± 0.54, 0.76 ± 0.61, and 1.23 ± 0.95, respectively. Significant difference is observed only in one direction (Rx, *p* = 0.03) in the paired setup errors obtaining from OSIS and CBCT, without significant differences in five directions.

**Conclusion:**

OSIS is a repeatable and reliable system that can be used to detect misalignments with accuracy, which is capable of supplementing CBCT for WBRT after BCS. We believe that an OSIS may be easier to use, quicker, and reduce overall dose as this method of patient alignment does not require ionizing radiation.

## 1 INTRODUCTION

Adjuvant radiotherapy is essential in patients with early breast cancer who require breast‐conserving surgery (BCS). Radiotherapy to the whole breast after such surgery results in good 5‐year local control, and a 5% increase in overall survival at 15 years.^1^ Correct target positioning plays a crucial role in accurate dose delivery in conformal radiotherapy and is much more important in intensity‐modulated radiotherapy (IMRT). Several factors can lead to uncertainties with regard to setup: variability in patient positioning, changes in breast shape, and breathing. A clinical target volume–planning target volume (CTV–PTV) margin is determined by setup errors according to clinical practice.^2^ The CTV–PTV margin may be decreased via image‐guided protocols for setup correction.^3^

Cone‐beam computed tomography (CBCT) has an important role in image‐guided breast radiotherapy because it can be used to detect variations in soft tissue.^4^ However CBCT increases the workload and also exposes patients to additional radiation.^5^ New imaging modalities such as fiducial markers inside the target volume monitored by portal imaging, ultrasound systems, and optical surface system of fast, noninvasive and nonradiation exposure have been investigated recently.^6^

An optical surface imaging system (OSIS) can be used to establish a three‐dimensional (3D) surface model using a laser without exposing the patient to additional radiation.^7^ Studies^8^, ^9^ have suggested that the setup correction could be calculated and utilized on the patients before irradiation by registering this surface model with that of a reference setup. Different optical systems include Catalyst system and Sentinel system and have been evaluated in different clinical settings. The high accuracy of such systems has been shown in several investigations.^8^, ^9^, ^10^, ^11^

We investigated an OSIS (Sentinel; C‐RAD Positioning, Uppsala, Sweden) in women who had whole‐breast radiotherapy (WBRT) after BCS. We compared the setup corrections determined by OSIS with those from CBCT carried out routinely.

## 2 MATERIALS AND METHODS

### 2.1 OSIS

**FIGURE 1:**
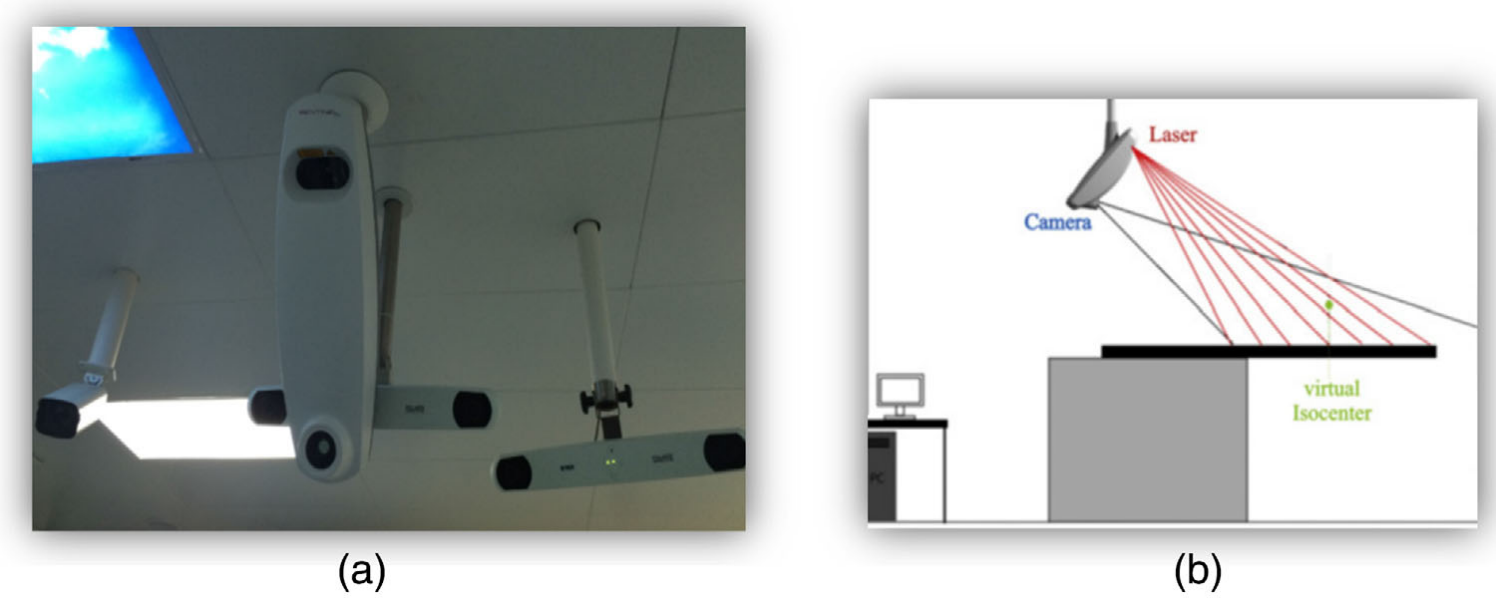
(a) Sentinel, C‐RAD laser, and camera, mounted in the ceiling in front of the gantry. (b) Sentinel, C‐RAD scanner is connected to the personal computer (PC) running the c4D software

Sentinel comprises a single scanner unit (which contains the laser and camera) mounted in the ceiling in front of the gantry (Figure 1). During surface acquisition, a laser line is swept along the chest of the patient, while the camera records a series of images. Sentinel can acquire more than 50 contours per second, and the patient surface, containing several hundred contours, is quickly captured in a few seconds. From the data acquired, the entire 3D surface of the patient can be reconstructed using laser‐line triangulation. The patient outline is extracted from the reference CT in the treatment planning system and used by OSIS for alignment. Sentinel registers the acquired surface with the reference surface via rigid‐body registration and reports translational and rotational setup corrections that can be applied by a 6D treatment couch. A CBCT system (Axesse; Elekta Instruments, Stockholm, Sweden) installed on the same treatment unit as the OSIS was considered as the reference for patient‐positioning system.

### 2.2 Clinical series

**FIGURE 2:**
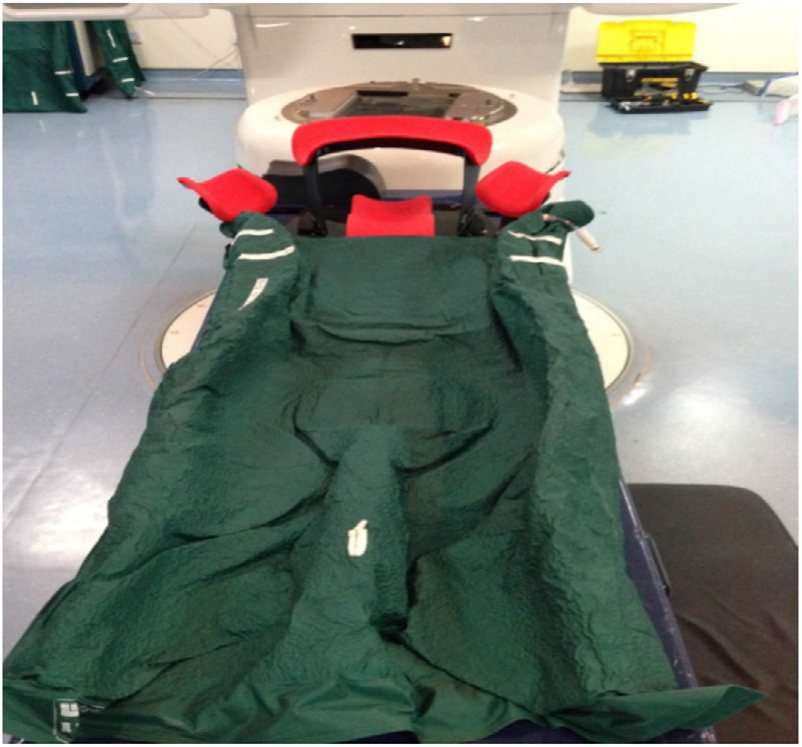
Breast posirest‐2 arm support (CIVCO, Kalona, USA) combined vacuum

Ten patients (mean 41 years old, range: 34–46) who underwent BCS for breast cancer were enrolled. Six of right and four of left breast. All patients diagnosed pT_1‐2_ N_0_M_0_ would accept whole breast irradiation. Patients underwent simulation by 16‐slice CT (Brilliance; Philips Amsterdam, the Netherlands) in the supine position free breathing with contiguous slices (thickness, 5 mm). The Posirest two‐arm support (Civco Medical Solutions, Kalona, IA, USA) combined with a vacuum (Figure 2) was used for patient setup. The delineation of target volume was segmented in the treatment planning system (Oncentra; Elekta) and used as the reference for CBCT. Area of interest, such as CTV, and organ at risk (OARs) (ipsilateral lung and heart) were defined. The CTV was defined as the entire breast tissue starting 5 mm below the skin. The PTV was defined by adding a 5‐mm margin to the CTV except the direction of the skin. IMRT using four fields to a total prescribing dose of 46 Gy in 23 fractionations was implemented in all cases. Subsequently, all patients received an electron‐boost dose of 14 Gy in seven fractionations to the surgical bed.

### 2.3 Data acquisition

**FIGURE 3:**
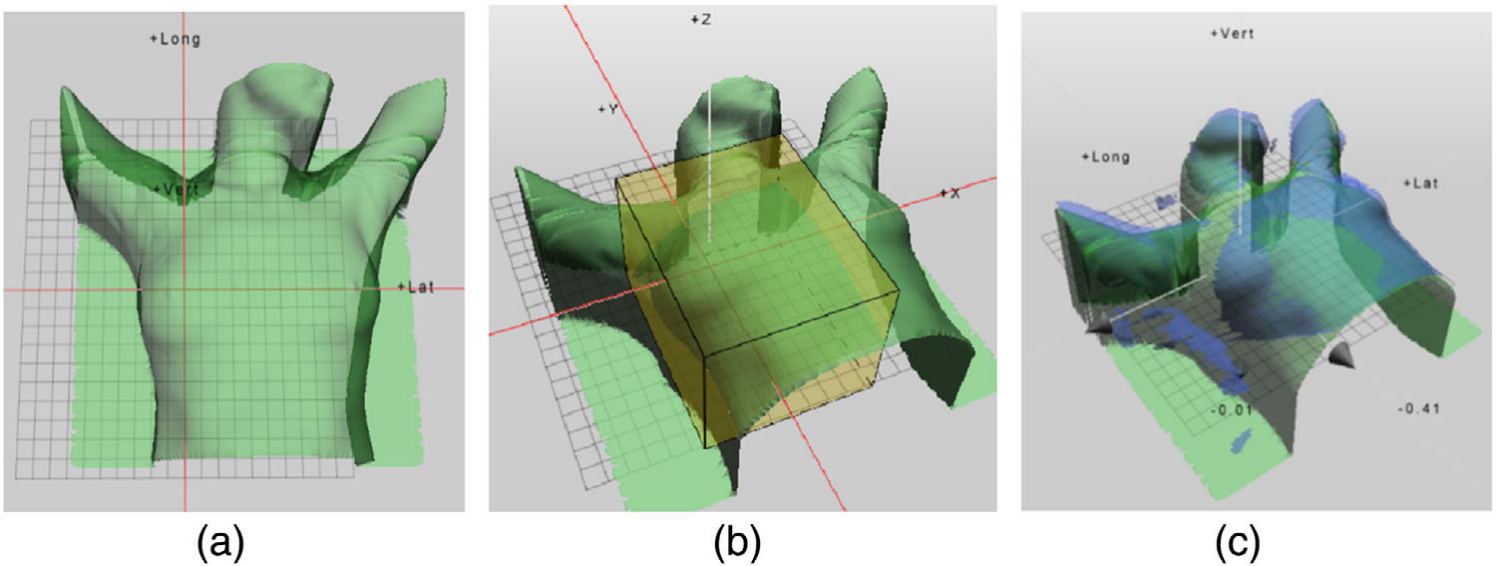
(a) Extracted from the planning CT system was used as Sentinel reference CTref. (b) Optical scan area range from the supraclavicularregion to submammary sulcus. (c) Images of surface registration: green is the reference contour surface, and blue is new contour surface obtained by laser scan

Registration data for 130 setups for 10 patients treated in the breast region were collected. We extracted the patient outline from the CT‐based treatment‐planning system, which was regarded as the reference for OSIS and CBCT (Figure 3a).

During the first treatment session, an optical surface image of the thorax (range of the scanning area was from the supraclavicular region to the submammary sulcus) (Figure 3b) was obtained and recorded by Sentinel. The surface used for registration was averaged over 4–5 scans during a total time of 5 s to account for breathing motion (Figure 3c). Setup errors in six directions (translational and rotational) were recorded. Simultaneously, thorax images from a routine CBCT scan were acquired compared with the treatment plan CTref. Automated registration based on soft tissue and bone markers was performed and then manually adjusted by the radiation physician. The patient was aligned to an optimal isocenter position by applying the 6D couch shifts based on CBCT to treatment plan CTref registration. And paired setup errors in the six directions were calculated. We detected position via the OSIS and CBCT for the first five fractionations of radiotherapy, and then twice a week.

### 2.4 Statistical analyses

The paired data were analyzed by the Student's *t*‐test on SPSS 18.0 software (IBM, Armonk, NY, USA).

### 2.5 Ethical approval of the study protocol

The study protocol was approved by the ethics committee of our hospital. All patients have signed informed consent to participate in our study.

## 3 RESULTS

**FIGURE 4:**
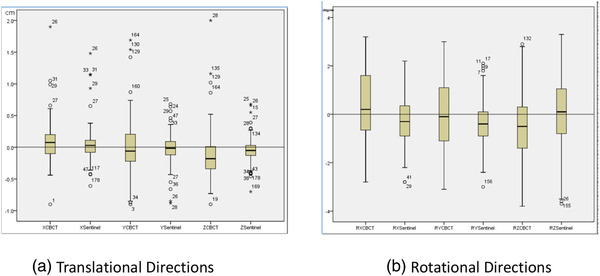
(a) Distribution of set up errors of translation respectively from cone‐beam computed tomography (CBCT) and sentinel. (b) Distribution of set up errors of rotation respectively from CBCT and sentinel

Setup errors of six directions along the main field axes from the OSIS and CBCT were calculated and shown as the mean ± SD (standard deviation) (Figure 4). The absolute translational setup error (mean ± SD) in x (lateral), y (longitudinal), and z (vertical) axes and rotational setup error (mean ± SD) in Rx (pitch), Ry (roll), and Rz (yaw) axes detected by both CBCT and OSIS before irradiation are shown in Table 1. There are no significant differences (*p* < 0.05) on the paired setup errors in five directions from the OSIS and CBCT: X (*t* = −1.827, *p* = 0.07), Y (0.125, 0.9), Z (1.595, 0.112), Ry (−1.717, 0.09), and Rz (2.382, 0.6). Significant difference was only shown in one direction: Rx (*t* = −3.409, *p* = 0.03)

**TABLE 1:** The setup errors (mean ± SD) in six directions detected by Sentinel and cone‐beam computed tomography (CBCT) prior radiation

Absolute set up errors of 6D direction (mean ± SD)						
	Lateral (cm)	Longitudinal (cm)	Vertical (cm)	Pitch Rx (°)	Roll Ry (°)	Yaw Rz (°)
CBCT (*n* = 130)	0.21 ± 0.21	0.29 ± 0.26	0.42 ± 0.22	0.83 ± 0.7	1.12 ± 0.79	1.07 ± 0.81
Sentinel (*n* = 130)	0.14 ± 0.18	0.15 ± 0.14	0.13 ± 0.13	0.77 ± 0.54	0.76 ± 0.61	1.23 ± 0.95
Difference of (Sentinel–CBCT)	0.06 ± 0.19	0.10 ± 0.18	0.23 ± 0.16	0.58 ± 0.48	0.55 ± 0.77	0.96 ± 0.73
T	−1.827	0.125	1.595	−3.409	−1.717	2.382
P	0.07	0.9	0.112	0.03*	0.09	0.6

^*^ significant difference.

## 4 DISCUSSION

Image‐guided radiotherapy (IGRT) has led to substantial improvements in the modern radiation oncology. The term “IGRT” refers to the use of various imaging methods to correct possible setup errors in patient position.^6^ In this study, we paid attention to position verification. Verification of 2D portal imaging ensures reproducibility within certain limits. Integrating various radiological techniques within the treatment room for guiding radiation delivery has greatly improved management of geometric uncertainties in radiotherapy and ushered in the paradigm of IGRT.^12^ The “gold standard” CBCT image set is based mainly on bony structures and soft tissue but has disadvantages^13^: the image quality of IGRT systems is obviously inferior to that of CT for diagnosis; the longer acquisition times of scans of IGRT systems gave rise to significant breathing artifacts; the extra dose delivered through daily imaging could increase the risk of a secondary malignancy.

IMRT, volumetric modulated arc therapy, and tomotherapy (TOMO) are options of postoperative WBRT combining with simultaneous local boost radiotherapy, or partial‐breast radiotherapy, but they need more strict verification of the target position. Indeed, the accuracy of positioning patients with breast cancer during radiotherapy is crucial. The reproducible positioning of the patient over the entire course of the radiotherapy is essential for receiving the planned doses of radiation and to decrease toxicity of OAR.^14^ For setup verification in breast‐cancer patients, electronic portal image devices (EPIDs) have been used to verify the tumor position by the bony anatomy and breast tissue. The visible structures in the image are mainly the sternum and ribs. The soft tissue in the breast is the main target of irradiation, but more accurate images/methods are needed to verify the soft‐tissue target. The major advantage of using CBCT instead of an EPID is the use of 3D reconstruction. However, the process of kV‐CBCT acquisition, reconstruction, and online registration takes a long time, and the cooperation of patients is necessary.^15^ kV‐CBCT acquisition can be approximated with three electronic portal images.^16^ Both EPIDs and CBCT are time‐consuming, especially in respiratory‐gated WBRT.^17^ Alignment in breast‐cancer patients based solely on bony anatomy can lead to inter‐fractional inconsistencies in the position of the breast surface, which may deform and change volume throughout treatment.^18^, ^19^ The potential benefits of surface imaging as an additional tool for patient positioning in various breast‐irradiation methods have been reported in several studies.^9^, ^20^, ^21^ The real time additional information on the shape and position of the breast was provided by surface imaging. Hence, we applied an OSIS (Sentinel) for WBRT, when a Sentinel image was considered as the reference. Wei et al.^11^ find that optical surface imaging by Sentinel has a significant correlation with CBCT in detecting setup errors in postoperative radiotherapy for breast cancer. Sentinel can be used to supplement to CBCT to avoid unnecessary imaging radiation dose to patients. In our study, no significant difference was seen in setup error between OSIS and CBCT in five directions. The significant difference was only shown in one direction: Rx (Pitch). Laser detection may be more sensitive in rotational direction than CBCT. While rotation setup error detection maybe further investigated, limitation of our study was that the study cohort was small.

3D surface imaging, in addition to CBCT, can aid patient positioning (especially for tumors near the body surface) and may complement (or partially replace) IGRT with X‐ray‐based volume imaging. The position verification for daily treatment is repeated by in‐room laser alignment to skin marks or fixation aids (e.g., thermoplastic films, vacuum bags). Pallotta et al.^7^ reported that, by comparing Sentinel with CBCT and portal imaging under measurements with a rigid phantom, the setup‐verification device in Sentinel was reproducible, consistent, and capable of detecting misalignments with precision better than 1 mm and 1°, and that Sentinel and CBCT were more accurate when compared with portal imaging. Several scholars have investigated the technical performance of an OSIS and found accuracies in the submillimeter range.^2^, ^7^ In our study, the absolute translational setup error (mean ± SD) in six directions detected by Sentinel before irradiation was less than 1.5 mm and 1.1°. The system precision was more favorable than 1.5  mm and 1.1° when a Sentinel image was considered as the reference.

In conclusion, Sentinel is a repeatable and reliable system that can be used to detect misalignments accurately, which is capable of supplementing CBCT for WBRT after BCS. We believe that an OSIS may be an efficacious supplement to standard CBCT for daily verification of tumor position.

## CONFLICT OF INTEREST

All authors declared that there are no conflict of interest to disclose.
